# Single Prolonged Stress as a Prospective Model for Posttraumatic Stress Disorder in Females

**DOI:** 10.3389/fnbeh.2019.00017

**Published:** 2019-02-11

**Authors:** Roxanna J. Nahvi, Chiso Nwokafor, Lidia I. Serova, Esther L. Sabban

**Affiliations:** Department of Biochemistry and Molecular Biology, New York Medical College, Valhalla, NY, United States

**Keywords:** anxiety, CRH, depression, females, locus coeruleus, mRNA, neuropeptide Y, tyrosine hydroxylase

## Abstract

Sex plays an important role in susceptibility to stress triggered disorders. Posttraumatic Stress disorder (PTSD), a debilitating psychiatric disorder developed after exposure to a traumatic event, is two times more prevalent in women than men. However, the vast majority of animal models of PTSD, including single prolonged stress (SPS), were performed mostly with males. Here, we evaluated SPS as an appropriate PTSD model for females in terms of anxiety, depressive symptoms and changes in gene expression in the noradrenergic system in the brain. In addition, we examined intranasal neuropeptide Y (NPY) as a possible treatment in females. Female rats were subjected to SPS and given either intranasal NPY or vehicle in two separate experiments. In the first experiment, stressed females were compared to unstressed controls on forced swim test (FST) and for levels of expression of several genes in the locus coeruleus (LC) 12 days after SPS exposure. Using a separate cohort of animals, experiment two examined stressed females and unstressed controls on the elevated plus maze (EPM) and LC gene expression 7 days after SPS stressors. SPS led to increased anxiety-like behavior on EPM and depressive-like behavior on FST. Following FST, the rats displayed elevated tyrosine hydroxylase (TH), CRHR1 and Y1R mRNA levels in the LC, consistent with increased activation of the noradrenergic system. The expression level of these mRNAs was unchanged following EPM, except Y1R. Intranasal NPY at the doses shown to be effective in males, did not prevent development of depressive or anxiety-like behavior or molecular changes in the LC. The results indicate that while SPS could be an appropriate PTSD model for females, sex differences, such as response to NPY, are important to consider.

## Introduction

Sex differences are prevalent in neuropsychiatric disorders. Men tend to be more susceptible to attention-deficit hyperactivity disorder and substance abuse while depression, eating and anxiety disorders are more prevalent in women (Hudson et al., 2007; Marcus et al., 2008; Bangasser and Valentino, 2014; Kucharska, 2017; Green et al., 2018). Moreover, women are at nearly double the risk for developing symptoms of posttraumatic stress disorder (PTSD) compared to men (Ditlevsen and Elklit, 2010). PTSD is a disabling, long-lasting, and difficult-to-treat neuropsychiatric disorder that develops in a subset of individuals after exposure to traumatic stress. Core symptomology of patients with PTSD are hyperarousal behavior, avoidance, re-experiencing of the trauma, and negative changes in cognition or mood (Friedman, 2013). It is often co-morbid with depression, drug abuse, alcoholism, increased risk of suicide, and marked psychosocial and occupational impairments.

Changes in the hypothalamus-pituitary-adrenal (HPA) axis and its regulators glucocorticoid receptor and FKBP5 and the noradrenergic system are strongly associated with PTSD symptoms (O’Donnell et al., 2004; Yehuda, 2005; Hendrickson and Raskind, 2016). The HPA axis is activated during stress releasing CRH from the hypothalamus to act on the anterior pituitary, which stimulates ACTH synthesis and release. ACTH then acts on the adrenal gland to synthesize and release glucocorticoids into circulation.

The catecholaminergic system, both peripherally and centrally, play key roles in responding to stress. Patients with PTSD have exaggerated noradrenergic activity, with elevated norepinephrine (NE) in the cerebral spinal fluid (CSF) correlating to PTSD severity (Geracioti et al., 2001). The locus coeruleus (LC), with its widespread afferents, is the major source of NE in the brain (Valentino and Van Bockstaele, 2008). In addition to responding to stress, the LC/NE regulates arousal, memory consolidation, emotion, and cognition, among other neurologic processes (Southwick et al., 1999; Kobayashi, 2001; Takeuchi et al., 2016).

A number of animal models of PTSD have been proposed and were used primarily with males (Cohen et al., 2012; Daskalakis et al., 2013; Goswami et al., 2013; Whitaker et al., 2014; Deslauriers et al., 2018). The single prolonged stress (SPS) paradigm is one of the best models for eliciting PTSD related symptoms in rodents such as anxiety, depression, hyperarousal, reduced social behavior, impaired fear extinction and cognition, as well as molecular changes in the HPA axis and NE system and evaluating possible pharmacologic interventions (Liberzon et al., 1997; Yamamoto et al., 2008, 2009; Eagle et al., 2013; Sabban et al., 2015b; Souza et al., 2017; Lisieski et al., 2018). However, few studies with SPS have included females (Keller et al., 2015; Pooley et al., 2018a,b).

Previous studies indicate that neuropeptide Y (NPY) has promise to provide therapeutic relief of PTSD symptoms in males (Serova et al., 2013, 2017; Laukova et al., 2014; Sabban et al., 2015a, b; Schmeltzer et al., 2016; Sabban and Serova, 2018; Sayed et al., 2018). NPY may be an important potential pharmacologic therapy in addition to the SSRIs paroxetine and sertraline, the only two drugs FDA approved for PTSD treatment which are not sufficiently effective (Alexander, 2012; Krystal et al., 2017). NPY is one of the most abundant endogenous peptides. It is involved in regulating many systems throughout the body including sleep, appetite, memory, anxiety, fear, and stress (Brothers and Wahlestedt, 2010; Reichmann and Holzer, 2016; Tasan et al., 2016; Kautz et al., 2017). Considerable evidence from humans and animals demonstrate an association between NPY and stress resilience (Morales-Medina et al., 2010; Thorsell, 2010; Enman et al., 2015; Kautz et al., 2017). Studies in soldiers revealed higher plasma NPY is associated with increased positive coping (Morgan et al., 2000; Yehuda et al., 2006). PTSD is associated with low plasma NPY levels and the severity of symptoms is negatively correlated with CSF NPY levels (Rasmusson et al., 2000; Morgan et al., 2003; Sah et al., 2009, 2014). NPY is proposed to provide resilience by controlling pro-stress transmitters CRH and NE (Kask et al., 2002; Heilig, 2004; Thorsell, 2010; Sah and Geracioti, 2013; Sabban et al., 2015a).

Selective delivery of NPY to the brain by intranasal administration is effective at early intervention and reversal of PTSD symptoms in male rats while avoiding peripheral side effects (Serova et al., 2013; Laukova et al., 2014; Sabban et al., 2015a; Camp et al., 2018). A recent clinical trial demonstrated high-dose intranasal NPY can reduce self-reported anxiety levels in PTSD patients (Sayed et al., 2018).

In this article, our primary goal was to evaluate SPS as an appropriate model for PTSD in female rodents in terms of anxiety, depressive symptoms and changes of gene expression in the LC. Our secondary aim was the assessment of intranasal NPY as a potential early intervention for females.

## Materials and Methods

### Animals

All experiments were performed in accordance with the PHS policy and NIH Guide for the Care and Use of Laboratory Animals. All animal studies were approved by the New York Medical College’s Institutional Animal Care and Use Committee (IACUC). The approved protocol number is 33-1-0517. Female Sprague-Dawley (SD) rats were purchased from Charles River (Wilmington, MA, USA). Animals were maintained on a 12-h light/dark cycle at 22°C with food and water *ad libitum*. They were housed four per cage for at least a week prior to the experiment.

### Single Prolonged Stress (SPS)

SPS was performed between 9 am and 2 pm as previously described (Serova et al., 2013). The rats were immobilized for 2 h on a metal board by taping the limbs with surgical tape and restricting the motion of their head. Then, they were immediately subjected to force swim in a plexiglass cylinder (50 cm height, 24 cm diameter; Stoelting, Wood Dale, IL, USA) filled two-thirds with 24°C fresh water. They were dried and allowed to recuperate for 15 min and then exposed to ether vapor in a bell jar until loss of consciousness. Afterwards, all animals were housed two per cage and left undisturbed until experimental testing.

### Intranasal NPY Administration

Rats were administered a single intranasal infusion of NPY (NeoBioSci, Cambridge, MA, USA) freshly dissolved in water, or equal volume of water (vehicle) while the animals were under the influence of ether (the last SPS stressor). A pipetteman with disposable plastic tip was used to infuse 10 μl into each nostril. Care was taken to avoid contact with the intranasal mucosa. Following intranasal administration, the head of the animal was held in a tilted back position for approximately 15 s to prevent loss of solution from the nostrils.

### Experiment 1: Forced Swim Test (FST) for Depressive Symptoms

**Figure 1:**
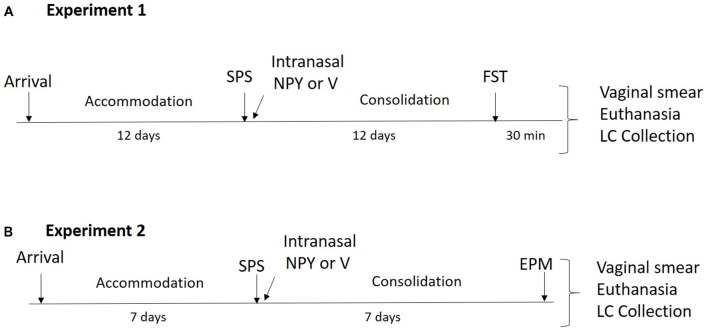
Design of the experiments. **(A)** In experiment 1, animals underwent a 12-day accommodation period upon arrival after which they were exposed to single prolonged stress (SPS) stressors and were administered 150 μg of intranasal neuropeptide Y (NPY) or vehicle while under the influence of ether. After a 12-day consolidation period, forced swim test (FST) was performed and vaginal smears, euthanasia and locus coeruleus (LC) collection occurred 30 min later. **(B)** In experiment 2, animals had a 7-day accommodation period after which they underwent SPS stressors and were administered 300 μg of intranasal NPY or vehicle while under the influence of ether. After a 7-day consolidation period, elevated plus maze (EPM) was performed and immediately afterwards vaginal smears, euthanasia and LC collection occurred.

The experimental design is shown in Figure 1A. Forty-eight 7-week-old, naturally cycling female SD rats (150–175 g) underwent a 12-day acclimation period and were randomly assigned to experimental or control groups (*n* = 16). Thirty-two animals underwent SPS and were infused with 150 μg intranasal NPY (*n* = 16) or vehicle (*n* = 16) immediately afterwards while still under the influence of ether. After the 12-day consolidation period, they were tested on the forced swim test (FST) together with an unstressed control group. The animals were euthanized 30 min after the FST, vaginal smears were collected and the LC region of the brain (9.2–10.4 mM posterior to the Bregma) was isolated and frozen immediately in liquid nitrogen for molecular analysis.

### Experiment 2: EPM for Anxiety Symptoms

The experimental design is shown in Figure 1B. Thirty-six female 7-week-old, naturally cycling SD rats (150–175 g) arrived at the animal facility and had a 1-week acclimation period. Twenty-four animals underwent SPS and were infused with 300 μg intranasal NPY (*n* = 12) or vehicle (*n* = 12) immediately afterwards. Rats were left undisturbed for 1 week and then tested on the elevated plus maze (EPM) together with unstressed controls (*n* = 12). The animals were euthanized immediately afterwards. Vaginal smears and LC punches were collected as described above.

### Forced Swim Test (FST)

FST was performed in plexiglass cylinders filled to two-thirds with 24°C fresh water for 5 min and behavior during the forced swim was videotaped as described by Serova et al. (2013). Time spent swimming, defined as movement of the forelimbs and hind limbs, and the time spent immobile when the animal showed no movement, or only movements needed to keep its head above the water was scored by a trained individual blinded to the experimental groups.

### Elevated Plus Maze (EPM)

Anxiety-like behavior was tested on the EPM as previously described (Serova et al., 2013). The apparatus (Stoelting, Wood Dale, IL, USA) 50 cm above ground level had four cross shaped platforms, two platforms with a 2-cm-high plexiglass wall were open while the other two platforms with 40-cm-high opaque walls on sides were closed. Arms of the same type were located opposite each other. Experiments were performed in a room with dim light. Animals were given 30 min to acclimate to the room prior to each experiment. Every rat was placed on the central platform with their head towards an open arm and allowed 5 min to explore the maze. The maze was cleaned with 70% ethanol between animals. The test was recorded using a tracking software (Viewer 3.0) Biobserve and the following measurements were taken; open arm (OA) and closed arm (CA) entries; total entries into all arms; duration of exploration in open and closed arms; time and frequency of risk assessment; number of head dips; and total distance traveled. Arm entry is defined as entering an arm with all four paws. Time in the arms was calculated as percent of the total time of the test. Percent of entries was calculated as percent of total open or CA entries to the total of all arm entries. Risk assessment is assessed by the rat poking its head or trunk into an OA while its hind quarters were located in one of the CA (Augustsson et al., 2005). We calculated anxiety index as 1 − [(time spent in OA/total time on the maze)/2 + (number of entries to the OA/total exploration on the maze)/2] (Cohen et al., 2012).

### Real-Time Polymerase Chain Reaction (PCR)

Total RNA from LC was isolated using STAT 60 (Tel-Test Inc., Friendswood, TX, USA). and concentration was quantified using Nano Drop 2000 (Thermo Fisher Scientific, Pittsburgh, PA, USA). Reverse transcription of RNA (500 ng) was performed with the RevertAid First Strand cDNA Synthesis kit (Thermo Fisher Scientific) according to the manufacturer’s protocol, using an oligo dT primer. For quantitative Real-Time Polymerase Chain Reaction (PCR), 2 μl of cDNA product was mixed with 12.5 μl of FastStart Universal SYBR Green Master Rox (Roche Diagnostics, Indianapolis, IN, USA) and 1 μl of the following primer pair sets: rat tyrosine hydroxylase (TH; forward 5′-CCGGTCTACTGTCCGCCCGT-3′, reverse 5′-TCATGGCAGCAGTCCGGCTC-3′), GAPDH (forward 5′-TGGACCACCCAGCCCAGCAAG-3′, reverse 5′-GGCCCCTCCTGTTGTTATGGGGT-3′), CRHR1 (Qiagen, cat. no. PPR44886F), NPY receptor 1 (Y1R; cat no. PPR6253024), or NPY receptor 2 (Y2R; cat no. PPR06816A) to a final volume of 25 μl, and analyzed on an ABI7900HT Real-Time PCR instrument (Applied Biosystems, Carlsbad, CA, USA). Each gene was normalized to GAPDH mRNA levels and expressed as the relative fold change vs. unstressed control, calculated using the ΔΔCt method (Livak and Schmittgen, 2001).

### Vaginal Smears

Vaginal smears were prepared according to the procedure outlined in McLean et al. (2012). In brief, a vaginal lavage was performed using distilled water and the collected specimen were allowed to dry on a glass slide. The slides were stained with 0.1% crystal violet and observed under a light microscope at 10× and 40× objectives to determine the phase of the estrus cycle by a trained researcher blind to the experiment conditions.

### Statistical Analysis

Data analysis was performed in Prizm 8 (GraphPad) software. Normality test was done using D’Agoustino and Pearson Omnibus. Data were analyzed by planned comparison *t*-test to evaluate the primary and secondary aims of this study. Outliers were removed when they were greater than two standard deviations away from the mean. Values at *p* ≤ 0.05 were considered significant.

## Results

### Experiment 1: FST for Depressive Symptoms

**Figure 2:**
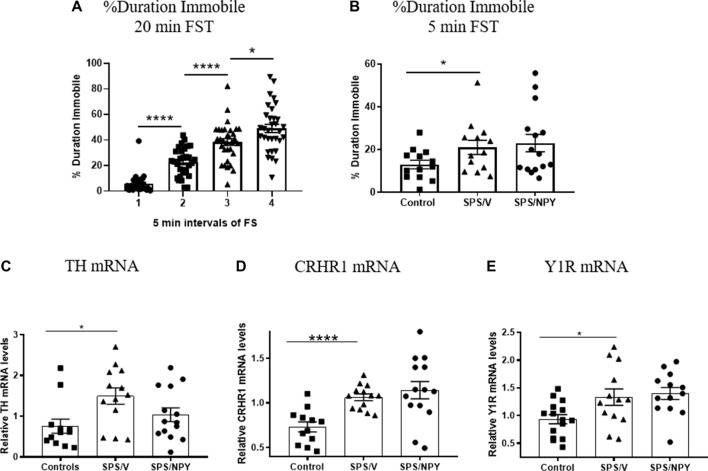
Effect of SPS on response to FST. Female rats were unstressed (Control), or exposed to SPS and while still under the influence of ether (the last stressor) administered 150 μg/rat of NPY; (SPS/NPY) or vehicle (SPS/V). The forced swim portion of the SPS stressors was recorded and immobility scored in 5 min intervals **(A)**. Twelve days later they tested for **(B)** immobility time on the FST and changes in mRNA levels of: **(C)** tyrosine hydroxylase (TH); **(D)** CRHR1; **(E)** NPY receptor 1 (Y1R) in the LC 30 min later. Means ± SEM are shown. Each point represents values for an individual animal. Planned comparisons *t*-test determined differences between groups. *p < 0.05; ****p < 0.0001.

Development of immobility during the forced swim portion of SPS stressors, as well as depressive-like behavior and molecular changes in the LC 12 days after SPS were evaluated in females (Figure 2). The animals became progressively more immobile in each 5 min block during the 20 min forced swim after a 2 h immobilization. Animals in the 2nd block spent significantly more time immobile than the 1st block (*t*_(62)_ = 7.3, *p* < 0.0001), spending almost 4× more time floating. The duration immobile also increased in the 3rd block compared to the 2nd (*t*_(62)_ = 4.9, *p* < 0.0001) and the 4th block compared to the 3rd (*t*_(62)_ = 2.5, *p* = 0.01; Figure 2A).

In the 5 min FST, the SPS/V group spent more time immobile than controls (*t*_(24)_ = 2.1, *p* = 0.05). Early intervention administration of intranasal NPY did not prevent development of this depressive-like behavior (Figure 2B).

Thirty minutes following FST, animals were euthanized and quantitative RT-PCR performed to determine expression levels of several genes involved in mediating the response of the LC/NE system to stress. A *t*-test revealed that mRNA levels of TH, the rate-limiting enzyme in NE biosynthesis, was changed with higher levels in the SPS/V than in controls (*t*_(23)_ = 2.8, *p* = 0.011). Intranasal NPY did not attenuate TH mRNA levels in the LC (Figure 2C).

The mRNA levels for CRHR1, the CRH receptor subtype expressed in the LC, was significantly elevated in SPS/V animals as compared to controls (*t*_(23)_ = 4.9, *p* < 0.0001). However, there were no differences between the SPS/V and SPS/NPY groups (Figure 2D).

Since the response to NPY differed from that in males (Sabban et al., 2015a), we assessed gene expression of the Y1 and Y2 receptor subtypes (Figure 2E). There were significant changes in the levels of Y1R mRNA between the SPS/V and control groups (*t*_(26)_ = 2.4, *p* = 0.02), but not Y2R mRNA (data not shown). Y1R mRNA levels were elevated in the SPS treated animals and were not reduced by early intervention with intranasal NPY.

### Experiment 2: EPM for Anxiety Symptoms

**Figure 3:**
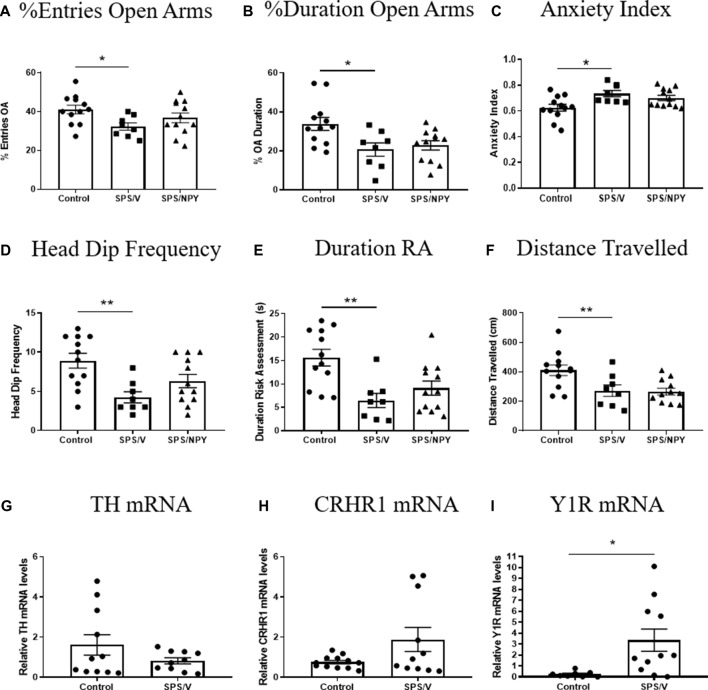
Effect of SPS on anxiety. Female rats were unstressed (Control), or exposed to SPS and while still under the influence of ether (the last stressor) administered 300 μg/rat of NPY (SPS/NPY) or vehicle (SPS/V). Seven days later they were tested on the EPM for: **(A)** entries into the open arms (OA); **(B)** duration in the OA; **(C)** anxiety index; **(D)** frequency of unprotected head dips; **(E)** total duration of risk assessment; **(F)** distance traveled; changes in mRNA levels of: **(G)** TH, **(H)** CRHR1, **(I)** Y1R in the LC immediately after EPM. Means ± SEM are shown. Each point represents values for an individual animal. Planned comparisons *t*-test determined differences between groups. *p < 0.05; **p < 0.01.

We examined anxiety behavior on the EPM and molecular changes in the LC 7 days after SPS in females (Figure 3). A *t*-test revealed significant differences in the percent of entries into the OA (*t*_(18)_ = 2.8, *p* = 0.01), with the SPS/V females entering the OA less frequently than unstressed controls. The mean of OA entries for SPS/NPY animals did not differ from the SPS/V group (Figure 3A). There were significant differences in the percent duration in OA between the SPS/V and unstressed control groups (*t*_(18)_ = 2.7, *p* = 0.02). SPS/V animals spent less time in the OA than controls, however intranasal NPY did not attenuate this behavior (Figure 3B). There were significant differences in anxiety index between the control and SPS/V groups. SPS/V females had a higher anxiety index than controls (*t*_(18)_ = 2.9, *p* = 0.01), but the SPS/NPY group did not differ significantly from SPS/V animals (Figure 3C).

There were significant differences in the frequency of unprotected head dips with SPS/V females having fewer unprotected head dips than controls (*t*_(18)_ = 3.6, *p* = 0.002). While there was a tendency for SPS/NPY animals to have more OA head dips than SPS/V, there were no significant difference between these groups (Figure 3D). There were significant differences in the total duration of risk assessment between the SPS/V and control groups (*t*_(18)_ = 3.7, *p* = 0.002). SPS/V females spent less time engaging in risk assessment behavior than control females while there was no difference with SPS/NPY animals (Figure 3E).

Furthermore, there were significant differences in total distance traveled (track length) between the SPS/V and control groups (*t*_(18)_ = 2.5, *p* = 0.02). Comparison between the means determined that SPS/V females traveled less distance during the EPM than control females. NPY treatment did not improve this behavior (Figure 3F).

Immediately following EPM, the LC was isolated and we performed quantitative RT-PCR to determine expression levels of several genes involved in mediating the response of the LC/NE system to stress. There were no observed significance differences among the groups in the mRNA levels of TH (*t*_(19)_ = 1.4, *p* = 0.17) and CRHR1 (*t*_(21)_ = 2.0, *p* = 0.06). However, Y1R levels differed with the SPS/V expressing elevated levels of Y1R mRNA compared to controls (*t*_(17)_ = 2.6, *p* = 0.02; Figures 3G–I).

## Discussion

The findings indicate that SPS could be an appropriate model for PTSD and associated disorders in females. SPS stressors triggered development of a number of PTSD-associated symptoms previously observed in males. During the SPS, the females exhibited progressively increasing immobility on the forced swim portion. SPS elicited elevated depressive-like behavior or passive coping strategy on FST, as well as anxiety and decreased risk assessment on EPM in females. Additionally, gene expression of TH, CRHR1, and Y1R in the LC/NE system was elevated following the FST and Y1R gene expression was elevated following EPM in the SPS-treated animals. However, intranasal NPY at concentrations effective for males had at most a marginal effect on these parameters in females.

### SPS as PTSD Model in Females

One of the important findings of this study is that animals subjected to SPS stressors exhibited elevated depressive-like behavior, or a passive coping strategy, and anxiety a week or more afterwards. Additionally, the animals demonstrated a continual increase in immobility throughout the 20 min forced swim stressor of the SPS. After each 5 min interval of force swim, the animals exhibited more floating behavior than the previous interval. The increased behavioral despair, as indicated by the immobility, suggests the prior immobilization stress and/or the forced swim stress appropriately creates a traumatic experience for the females.

This is the first study to evaluate depressive-like behavior on the FST after exposure to SPS in females. SPS/V females demonstrated more depressive-like behavior than unstressed controls 12 days after SPS. Recent discussions posit that the FST measures coping strategies and adaptation to stress rather than depression. In this theory, animals adapt to the forced swim stress by switching from active coping (swimming and climbing) to passive coping (floating; de Kloet and Molendijk, 2016). The FST has been implicated in many animal models of different psychiatric illnesses, suggesting depression might not be the sole behavioral condition involved (Commons et al., 2017). Nevertheless, our results add to growing evidence suggesting SPS elicits a depressive phenotype in females.

Previously, SPS was reported to trigger depressive measures of anhedonia in females, as assessed by reduced sucrose preference, and social interaction (Pooley et al., 2018b). Additionally, the dexamethasone suppression test showed decreased suppression of plasma corticosterone in females 1 week after SPS stressors (Pooley et al., 2018a,b). These measures, in conjunction with the results from this study, indicate that SPS is successful at eliciting a depressive phenotype.

Across all EPM behavioral measures, the stressed females showed more anxious behavior than unstressed controls 1 week after exposure to SPS stressors. SPS/V females spent less time and had fewer entries into the OA, more time and fewer entries into the CA, and higher anxiety index in the EPM than controls. Thus, the data suggests SPS successfully elicits anxiety in females as previously observed by Fan et al. (2013). Several other measures on the EPM altered in males by SPS were also observed in females, including reduced risk assessment, head dips, and locomotion (track length).

The reduced locomotion likely does not account for the increased anxiety on the EPM. In males, we have previously shown that NPY infusion alleviates anxiety as measured by the percent of open and CA entries and duration and anxiety index, however the track length (or distance traveled) remains unchanged as compared to animals infused with vehicle and lower than unstressed controls (Serova et al., 2013, 2014). Furthermore, the total arm entry between the three groups here did not differ significantly demonstrating changes in locomotion did not influence the number of arm entries.

Other models of PTSD have also been shown to elicit increased anxiety in females. Female rodents exposed to a cat (predator stressed) or cat or fox odor (predator scent) for 10 min exhibited anxiety-like behavior on the EPM 1 week later (Adamec et al., 2006; Mazor et al., 2009). However, these models are highly dependent on odor. Females have a more sensitive sense of smell than males even in humans (Bengtsson et al., 2001; Brand and Millot, 2001; Kass et al., 2017), and smell is a particularly prominent sensory feature in rodents. In contrast, SPS does not include an element of odor but nevertheless elicited many features of anxiety and may be more easily translatable to humans.

Several other behavioral phenotypes were also previously assessed in females following SPS with mixed results. These included: fear conditioning, hyperarousal and social interaction. The effect of fear conditioning is unclear. In one study, females exhibited more contextual freezing as compared to unstressed controls 2 weeks after SPS stressors (Fan et al., 2013). In contrast, Keller et al. (2015) indicated that SPS may not be appropriate for females since, in contrast to males, they did not display enhanced recall of extinguished fear 1 week after SPS. However, darting response (as opposed to freezing) during fear conditioning may be a more appropriate behavioral measure for females (Gruene et al., 2015).

The acoustic startle response was not changed in females 7 or 10 days after SPS indicating it did not trigger hyperarousal (Pooley et al., 2018a,b). SPS affected social interaction in females, but was dependent on housing of the animals (Pooley et al., 2018b).

Further support for SPS as an appropriate model for PTSD in females comes from the molecular changes seen here in the LC. In response to stress, hypothalamic and extrahypothalamic CRH is released and binds to CRHR1 in the LC, causing a more tonic phase in these cells and consequential NE release, increased vigilance, hyperarousal and anxiety (Valentino and Foote, 1987, 1988; Van Bockstaele et al., 1996; Page and Abercrombie, 1999; Valentino and Van Bockstaele, 2008). In fact, anxiety symptoms are prevented when the LC is inhibited optogenetically suggesting its crucial role (McCall et al., 2015).

There were pronounced changes in LC mRNA levels in SPS-treated animals after the FST, but only Y1R mRNA levels differed after the EPM. Thirty minutes following FST, there was an elevation of mRNA levels of TH, the rate limiting enzyme for NE biosynthesis, in the SPS/V group compared to previously unstressed controls, as observed in males (Serova et al., 2013). Given that the LC is the primary source of NE in the forebrain and the sole source of NE in the cortex and hippocampus, this likely leads to enhanced noradrenergic activity. In addition to TH, there was elevated LC gene expression of CRHR1 and Y1R in the females after the FST.

The changes in mRNA levels of Y1R in the LC following FST and EPM between unstressed controls and the SPS/V group suggest the involvement of Y1R and the NPY system in the female stress reaction. Y1R knockout (Y1R^−/−^) female mice exhibited anxiolytic or decreased depressive-like behavior 1 week after forced swim or EPM stress, as compared to Y1R wildtype controls exposed to the same stress (Painsipp et al., 2010). On the Morris water maze, females containing Y1R^−/−^ Y5R-expressing neurons perform better than controls (Longo et al., 2014). Furthermore after 1-h restraint, Y1R levels are increased 6 h later in the PVN and amygdala in males (Mele et al., 2004). Thus, the regulation of NPY and its receptors is implicated in orchestrating an appropriate stress response.

The same changes following FST in LC mRNA levels of TH and CRHR1 between stressed and unstressed females were not seen following the EPM. A limitation of this study is that all the animals underwent the stress of the behavioral testing. It is unclear if the mRNA levels seen are due to the SPS stressors or the behavioral experiment, as there were no stressed controls not exposed to behavioral testing in this study. Further studies with stressed controls are needed in order to ascertain if the molecular changes in the LC occur as a result of SPS. The stronger molecular changes in the FST experiment may be attributed to sensitivity of a reexperiencing effect, one of the main components of PTSD. The LC is highly implicated in the re-experiencing reaction and can contribute to larger NE responses (Elzinga and Bremner, 2002; George et al., 2013). Thus, the SPS-treated animals would have a surge of LC activation following a re-experiencing event, such as the FST.

Other molecular changes have been shown previously in females 1 week after SPS, further suggesting the strength of this model. Females subjected to SPS had increased cFos expression in the mPFC and amygdala, as compared to unstressed controls (Pooley et al., 2018a). Additionally, upregulation of GR in the hippocampus was found 1 week after SPS stressors (Keller et al., 2015). Interestingly, SPS stressors, but not predator stress, decreased GR expression in the PVN (Pooley et al., 2018a).

There is substantial evidence pointing to the conclusion that SPS produces significant and sufficient stress in females to elicit behaviors associated with PTSD. The impact of the stress is seen as early as in the forced swim portion of the SPS, with females exhibiting progressively more helplessness or passive coping behaviors. While this stress sufficiently provokes PTSD behavior, comparison to vehicle-treated males from one of our previous studies suggest it might be less severe for females. After 10 min forced swim during the SPS stressors, males spent over 90 percent of the time immobile compared to less than 50% of the time spent immobile by females. Throughout the 20 min forced swim after the 2 h immobilization, it appears females spent less time immobile in each 5 min block than males. The suggested differences could indicate that the SPS immobilization and/or forced swim stressors are milder for females. During the 5 min FST 1 week or more after SPS, it appears stressed females and unstressed controls spent less time immobile in FST, as compared to males. (Serova et al., 2013). Therefore, adjustments to the SPS paradigm might be needed to extend the same degree of stress males experience to females.

### Early Intervention With Neuropeptide Y in Females

The action of NPY is mediated by a family of G-protein coupled receptors including Y1R, Y2R, Y4R and Y5R. Y1R, a postsynaptic receptor, has been shown to be important in mediating the anxiolytic effects of NPY (Theisen et al., 2018). The Y2 receptor, located both pre- and post-synaptically, appears to be important in modulating activity of LC neurons (Kask et al., 1998). In this study, we observed increased Y1R, but not Y2R, gene expression in the SPS-treated female animals.

While our previous studies demonstrated the effectiveness of intranasal NPY to prevent development of PTSD-associated behaviors and molecular changes for male rats (Serova et al., 2013, 2017; Sabban et al., 2015a, b; Tasan et al., 2016), it appears that intranasal NPY may not be an effective treatment for females at the same dosage. NPY tended to reduce the increase in TH mRNA levels in the SPS/NPY group, but it was not significantly different from the SPS/V group. In the second experiment, a higher NPY dose was chosen to evaluate an observable therapeutic effect at higher concentrations. With the higher dose, there tended to be a slight improvement on some of the EPM measures, such as OA and CA entries, anxiety index, and frequency of head dips. Further studies are needed to determine if higher doses of NPY might be required for females.

NPY has been proposed to antagonize the actions of CRH (Schmeltzer et al., 2016). Given that females have increased sensitivity to CRH in the LC, a larger dose of NPY might be needed to antagonize its effect than in males (Heilig, 2004; Valentino and Bangasser, 2016; Bangasser et al., 2018). This increased sensitivity to CRH may be due to decreased internalization of the CRHR1 during stress, increased LC dendrite density, and increased CRHR-G_s_ coupling at baseline in cortical tissue and the LC, as compared to males (Bangasser et al., 2010, 2013). The molecular signaling and trafficking of the CRHR1 provides evidence of an impaired adaptation to stress in females.

With females, it is important to take into account the estrus cycle and influence of sex hormones. Estrogen has a protective effect on many of the symptoms of PTSD in rodents and humans (Contreras et al., 2000; Marcondes et al., 2001; Almeida et al., 2005; Serova et al., 2005; Milad et al., 2009; Kornstein et al., 2010; Young and Korszun, 2010; Bryant et al., 2011; Glover et al., 2012; Newhouse and Albert, 2015; Molina-Jímenez et al., 2017). Additionally, fluctuations in NPY receptors due to the estrous cycle have been reported, with increased hypothalamic Y1R expression in proestrus females compared to other phases of the estrous cycle (Martini et al., 2011). Estrogen response elements flank the Y1R gene and changes the gene expression as estradiol levels fluctuate (Eva et al., 2006). Furthermore, estrogen increases NPY-immunostaining neurons and NPY release in the hippocampus (Velíšková et al., 2015).

Here, vaginal smears were taken at sacrifice. However, only a limited number of animals were in each phase of the estrous cycle within each group. Future research conducted with females would require a larger cohort to accurately analyze SPS effects and NPY treatment on behavior in a phase-controlled manner.

## Limitations

There are some limitations to note regarding this study. The difference in acclimation and consolidation periods between the two experiments, due to uncontrollable circumstances, may complicate the interpretation of the results. The lack of stressed controls not exposed to behavioral testing limits the interpretation of the molecular changes post-SPS, as the behavioral testing could induce molecular changes itself. Furthermore, the lack of unstressed female control animals administered NPY serves as a limitation to the understanding of the behavioral and molecular effect intranasally administered NPY can have in an unstressed, female animal. In males, there was no effect of NPY in unstressed controls.

## Conclusion

Overall the results indicate that SPS, perhaps with some modifications, could be an appropriate model for PTSD and associated disorders in females. The diversity of symptoms makes finding a complete model for PTSD difficult. PTSD can manifest as depressive symptoms, anxiety, hyperarousal, social apathy, impaired cognition and altered mood. SPS in female rats elicits depressive symptoms, anxiety, altered social interaction, impaired cognitive processes as shown by fear conditioning, and ensuing molecular changes. Thus, it appears that SPS may provide a broad spectrum of PTSD impairments in female rodents, although they did not benefit from intranasal NPY treatment at the same doses given to males. As this is one of few articles investigating females post-SPS, there is a dire need to continue research efforts in this area.

## Data Availability

The datasets generated for this study are available on request to the corresponding author.

## Author Contributions

ES conceived and supervised the study, all authors participated in the study design. CN, RN and LS performed the experiments. RN, CN, LS performed the data analysis. RN, CN wrote the manuscript. All authors read and approved the manuscript.

## Conflict of Interest Statement

The authors declare that the research was conducted in the absence of any commercial or financial relationships that could be construed as a potential conflict of interest.
